# Impact of Inflammatory Cytokine Gene Polymorphisms on Developing Acute Graft-versus-Host Disease in Children Undergoing Allogeneic Hematopoietic Stem Cell Transplantation

**DOI:** 10.1155/2015/248264

**Published:** 2015-04-20

**Authors:** Riccardo Masetti, Daniele Zama, Milena Urbini, Annalisa Astolfi, Virginia Libri, Francesca Vendemini, William Morello, Roberto Rondelli, Arcangelo Prete, Andrea Pession

**Affiliations:** ^1^Pediatric Oncology and Hematology Unit “Lalla Seràgnoli”, Department of Pediatrics, University of Bologna Sant'Orsola-Malpighi Hospital, No. 11, 40138 Bologna, Italy; ^2^“G. Prodi” Cancer Research Center, University of Bologna, Bologna, Italy

## Abstract

Single nucleotide polymorphisms (SNPs) in gene encoding pro- and anti-inflammatory factors have been associated with the occurrence of aGvHD. We retrospectively tested a wide panel of 38 polymorphisms in 19 immunoregulatory genes, aiming to first establish, in a pediatric HSCT setting, which SNPs were significantly associated with the development of aGvHD. A significant association was found between aGvHD grades II–IV and SNPs of donor IL10-1082GG, and Fas-670CC + CT and recipient IL18-607 TT + TG genotype. aGvHD grades III-IV resulted associated with donor IL10-1082GG, Fas-670CC + CT, and TLR4-3612TT as well as the use of peripheral CD34+ cells as stem cell source. The multivariate analysis confirmed the association between donor IL10-1082GG and Fas-670CC + CT and aGvHD grades II–IV and between donor IL10-1082GG and TLR4-3612TT and aGvHD grades III-IV. In conclusion we found an association between IL10, FAS, and TLR4 in the donor and IL18 in the recipient and an increased risk of developing aGvHD in transplanted children. Knowledge of the SNPs of cytokine genes associated with aGvHD represents a useful tool for an integrated pretransplantation risk assessment and could guide the physicians to an optimal and more accurate HSCT planning.

## 1. Introduction

Acute graft-versus-host disease (aGvHD) remains one of the major determinants of the early outcome of allogeneic hematopoietic stem cell transplantation (HSCT). Disparities in human leukocyte antigen (HLA) molecules between the donor and recipient represent a crucial factor determining the alloresponse of aGvHD. Recent evidence has highlighted the role of donor and recipient single-nucleotide polymorphisms (SNPs) in genes encoding inflammatory factors in the occurrence of aGvHD [1]. Although this association has been identified and confirmed in many studies conducted in adults, little is known about cytokine gene SNPs and the onset of aGvHD in paediatric patients [2–6].

Moreover, most studies have focused on the association of a unique SNP or few SNPs with aGvHD, whereas few studies have broadly tested a composite group of polymorphisms [1]. We retrospectively studied a wide panel of cytokine gene SNPs with a known role in aGvHD pathogenesis and analysed the association of these SNPs with the incidence and grade of aGvHD. The aim of this study was to establish which SNPs among the panel of candidate SNPs play a significant role in the development of aGvHD in a paediatric HSCT setting.

## 2. Material and Methods

The entire study population consisted of 117 children (mean age: 9.5 (1–18) years) who underwent allogeneic HSCT in the Paediatric Haematology-Oncology Department of Bologna University between 1995 and 2010 and their respective donors (age: 26 (0–53) years). All patient records/information was anonymized and deidentified prior to analysis and all participants to the study also signed a written informed consent. The characteristics of the cohort are summarised in Table 1.

For sibling transplants, HLA typing was performed serologically or by low-resolution molecular typing. Unrelated patient-donor pairs were matched by high-resolution DNA typing. aGvHD was graded according to previously published criteria [7]. Genomic DNA was extracted using a QIAamp DNA Mini Kit-QIAGEN (Milan, Italy), and genotyping was performed using iPLEX Gold technology and MassARRAY high-throughput DNA analysis (Sequenom, Inc., CA). Briefly, the assay consists of an initial locus-specific PCR reaction, followed by single base extension using mass-modified dideoxynucleotide terminators of an oligonucleotide primer which anneals immediately upstream to the polymorphic site of interest. Using MALDI-TOF mass spectrometry, the distinct mass of the extended primer identifies the SNP allele. Specific assays were designed for the locus-specific amplification. In total, 38 SNPs in 19 immunoregulatory genes related to aGvHD onset risk (*ESR1, FAS, FCGR2A, IL1A, IL1B, IL2, IL6, IL10, IL10RB, IL18, MBL2, MTHFR, NOD2, TGFB1, TGFBR2, TLR4, TNF, TNFRSF1B,* and* VDR*) were selected. A complete list of the tested SNPs is reported in Supporting Table 1 in the Supplementary Material available online at http://dx.doi.org/10.1155/2014/248264. All alleles were in Hardy-Weinberg equilibrium, and the allele frequencies were not different between donors and recipients. SNPs with allele frequencies less than 0.15 were retained for analysis. An analysis of the association between SNPs, aGvHD, and clinical variables was performed using binary logistic regression methods and 3 different genetic models, including codominant, dominant, and recessive models (Table 1). The sample dimension was estimated assuming a frequency of the predisposing allele of 0.15, a disease prevalence of 0.3, and a genotype relative risk of 2.2 (for both the homozygote and the heterozygote conditions). Under these conditions, it is possible to identify a susceptibility allele with a statistical power of 80% and a type I error rate of 0.05 in a study of 117 individuals (cases and controls).

## 3. Results

In total, 48/117 and 13/117 patients had grades II–IV or III-IV aGvHD, respectively. The cumulative incidence (CI) of aGvHD in the analysed cohort was 57.6% globally and 41.7% and 10.5% for grades II–IV and III-IV, respectively. This CI of aGvHD is comparable to the value previously published in the paediatric field for grades III-IV, whereas this CI is slightly higher than that previously reported for grades II–IV [7].

The univariate analysis conducted on clinical variables showed a significant association between the source of stem cells (SSC), particularly peripheral blood stem cells (PBSCs), and severe grades III-IV aGvHD (*P* = 0.003).

Considering the cytokine gene SNPs with respect to donor genotype, a significant association was found in the univariate analysis between IL10-1082GG and FAS-670CC + CT and grades II–IV aGvHD (*P* = 0.012 and *P* = 0.027, resp.) or grades III-IV aGvHD (both *P* = 0.029). The TLR4-3612TT donor genotype was associated only with grades III-IV aGvHD (*P* = 0.002). Regarding the recipient genotype, IL18-607TT + TG was found to be associated with grades II–IV aGvHD (*P* = 0.014).

The multivariate analysis confirmed the association between grades II–IV aGvHD and the SSC, particularly PBSCs (Table 2; *P* = 0.016) and between grades II–IV aGvHD and the donor IL10-1082GG and FAS-670CC + CT genotypes (*P* = 0.08 and *P* = 0.029, resp.). Grades III-IV aGvHD was associated with donor IL10-1082GG (*P* = 0.057) and TLR4-3612TT (*P* = 0.002).

The probabilities of overall survival (OS) and event-free survival (EFS) were estimated using the Kaplan-Meier method. No difference in outcome was noted between patients carrying SNPs associated with aGvHD and patients without SNPs associated with aGvHD.

## 4. Discussion

This analysis of a large panel of 38 candidate SNPs in cytokine genes revealed that donor SNPs in* IL10*,* FAS,* and* TLR4* yield a greater risk for developing aGvHD. To our knowledge, this is the first attempt to simultaneously test such a large number of published genetic associations using a high-throughput technique in a relatively large cohort of paediatric transplant patients and donors.

The CI of aGvHD grades III-IV in our retrospective cohort was comparable to the value previously published in the paediatric field, whereas our CI was slightly higher than that previously reported for grades II–IV [7]. It remains relatively difficult to compare data on the incidence of aGvHD in a retrospective mixed cohort of paediatric patients who received donations following different forms of HLA matching (typed in different ways) or were administered different aGvHD prophylaxis regimens over a wide period of time (nearly 15 years).

We found that the only clinical variable that was significantly associated with an increased risk of aGvHD was the SSC, particularly PBSCs. Historically conflicting results have been reported regarding the incidence of aGvHD using different SSCs, such as PBSCs and bone marrow, although a recent meta-analysis clearly demonstrated a slightly but significantly higher risk of developing aGvHD and extensive chronic GvHD in patients receiving PBSCs [8].

IL10 is an immunomodulatory cytokine produced by B cells, regulatory T cells, monocytes, and dendritic cells that suppresses proinflammatory cytokine production such as TNF-*α*, IL-1A, IL-1B, IL-6, IL-12, and IFN-*γ*. SNPs in the promoter region of* IL10* assemble into 3 conserved haplotypes that lie between −1082 and −592: GCC, ATA, and ACC [9, 10]. Conflicting results have been reported regarding the genetic control of IL10 production, with certain authors describing an association between the increased production of IL10 and the GCC haplotype [9] and others reporting the same haplotype to be associated with decreased production [10]. Also in vivo the role of the polymorphisms in the promoter region of IL10 has been extensively studied with contrasting results by different authors [1, 11]. In particular the seminal studies of Lin et al. highlighted the synergistic effect between the IL10 genotype of the patient and the IL10 receptor *β* chain genotype of the donor. But the same authors found also a trend for an association of severe GVHD with the IL10/-592 genotype of donor. These observations well describe the importance and the complexity of the pathway of IL10 in the risk of developing GvHD in transplanted patients [4, 12]. In this context our data showed a correlation between donor IL10-1082GG and an increased risk of any grade of aGvHD. One possible explanation could be that this SNP may lead to a decrease in IL10 secretion, resulting in an increased alloreactivity of donor T cells. However, it is difficult to confirm this speculation in the absence of IL10 serum measurements, which was due to the retrospective nature of our study.

The FAS-670G SNP in donors was also correlated with aGvHD, confirming a previous observation of Mullighan et al. in a study performed in transplanted adults [13]. FAS-mediated apoptotic cell death is an important pathway involved in tissue damage in aGvHD. The FAS-670G SNP lies in a gamma-activated sequence response element in the FAS enhancer [14]. Although data regarding the functional consequences of this polymorphism are limited, the -670G polymorphism may increase FAS expression and thus mediate increased tissue damage during aGvHD by apoptotic cell death. Alternatively, this variant may influence the degree of apoptosis of donor lymphocytes, which has been shown to modulate GvHD in murine models [15].

Toll-like receptors are transmembrane proteins in immune cells with conserved molecular motifs. TLR-4 has been identified as a signal-transducing component in the lipopolysaccharide receptor complex that plays a crucial role in the pathogenesis of aGvHD [16, 17]. TLR-4 genetic variants have been mainly investigated in relation to infection susceptibility [18], and few data have been reported regarding their correlation with aGvHD [19]. In our paediatric cohort, we found that donor TLR4-3612TT increased the risk of aGvHD. One possible explanation for this finding is that this SNP could result in higher expression of TLR-4, leading to irregular LPS responsiveness, which may mediate increased inflammation via the transduction of LPS signals.

IL18 is a proinflammatory cytokine, elevated in patients with aGvHD, that induces Th1 differentiation and cytotoxic T-lymphocyte function. Patient IL18 GCG haplotype has been associated with improved survival and decreased transplant-related mortality after unrelated-donor bone marrow transplantation, while no association has been found between the occurrence of aGvHD and patient/donor haplotypes [20].

In our data, recipients' IL18-607TT + TG SNP and grades II–IV aGvHD have been also associated, even if the multivariate analysis did not confirm the significance of this association. Therefore, this data should be carefully evaluated and further studies should be performed to confirm that an increased risk of GvHD is linked to the SNPs of IL18.

Unlike our finding, SNPs on the promoter of other immunoregulatory genes, such as IL2, IL6, or MTHFR, have been described to be associated with aGvHD. These associations were not found in our pediatric cohort [6, 11, 21, 22]. This fact may possibly be explained by the limitations of our study that include the limited selection of candidate genes and genotypes and the heterogeneity of the study population owing to the requirement of a large number of patients. Also, we included patients with heterogeneous diagnoses receiving diverse conditioning regimens and various GVHD prophylaxes. Nevertheless this is the first attempt to test such a large number of published genetic associations in a large cohort of pediatric patients.

## 5. Conclusion

Donor SNPs in* IL10*,* FAS,* and* TLR4* seem to be significantly associated with aGvHD in a paediatric HSCT setting. Further insight into the mechanisms underlying the association between the single-gene SNPs could prompt new strategies for modulating the intensity of the alloimmune response and reducing the toxicity of aGvHD.

## Supplementary Material

SNPs genotyped and minor allele frequencies (MAF) in donors and recipients.

## Figures and Tables

**Table 1:** Association between clinical variable and aGvHD.

	Tot. = 117	%	GVHD I–IV			GVHD II–IV			GVHD III-IV		
			Tot. = 73	%	*P*	Tot. = 48	%	*P *	Tot. = 13	%	*P*
Sex											
Recipient											
M	82	70.0%	48	58.5%	*0.21 *	32	39.0%	*0.54 *	7	8.5%	*0.20 *
F	35	30.0%	25	71.4%		16	45.7%		6	17.1%	
Donor											
M	73	62.3%	47	64.3%	*1.00 *	31	42.4%	*0.70 *	9	12.3%	*0.76 *
F	44	37.7%	26	59.0%		17	38.6%		4	9.0%	
Underlying disease											
Leukemia and lymphoma	81	69.2%	50	61.7%	*0.33 *	36	44.4%	*0.47 *	8	9.8%	*0.28 *
Solid tumors	17	14.5%	13	76.4%		5	29.4%		1	5.8%	
Nononcologic disease	19	16.3%	10	52.6%		7	36.8%		4	21.0%	
Transplantation type											
Matched, related	43	36.8%	25	58.1%	*0.74 *	17	39.5%	*0.91 *	4	9.3%	*0.37 *
Matched, unrelated	47	63.8%	30	63.8%		20	42.5%		4	8.5%	
Mismatch, unrelated	27	23.0%	18	66.6%		12	44.4%		5	18.5%	
Conditioning regimen											
TBI-based	22	18.9%	16	72.7%	*0.33 *	13	59.0%	*0.10 *	3	13.6%	*0.70 *
BU-based	95	81.1%	57	60.0%		35	36.8%		10	10.5%	
Stem cells sources											
Bone marrow	92	78.6%	60	65.2%	0.*025 *	41	44.5%	0.*17 *	7	7.6%	0.003^*^
PBSC	25	21.4%	13	52.0%		7	28.0%		6	24.0%	
GVHD prophylaxis											
CSA only	33	28.2%	19	57.5%	*0.52 *	11	33.3%	*0.30 *	2	6.0%	*0.34 *
CSA + other	84	71.8%	54	64.2%		37	44.0%		11	13.0%	
Number of HSCT											
1	101	86.3%	62	61.3%	*0.78 *	40	39.6%	*0.58 *	11	10.8%	*1.00 *
>1	16	23.7%	11	68.5%		8	50.0%		2	12.5%	

M: male; F: female; yrs: years; TBI: total body irradiation; BU: busulfan; GVHD: graft versus host disease; CSA: cyclosporine A; PBSC: peripheral blood stem cells; ^*^ *P* < 0.05.

**Table 2:** Univariate and multivariate analysis of the variables significantly associated with severe GvHD.

	aGvHD	*n* with outcome/*n* with variant	*n* with outcome/*n* without variant	Univariate			Multivariate		
				ODDS ratio	CI 95%	*P*	ODDS ratio	CI 95%	*P*
IL10-1082GG DNR	II–IV	12/16 (75.0%)	33/85 (38.8%)	3.77	1.33–12.0	**0.012**	4.50	1.45–16.58	**0.008**
	III-IV	4/16 (25.0%)	7/85 (8.2%)	4.21	1.17–14.65	**0.029**	5.15	0.95–30.15	**0.057**

FAS-670CC + CT DNR	II–IV	38/74 (52.3%)	7/27 (25.9%)	2.81	1.12–7.65	**0.027**	2.90	1.11–8.35	**0.029**
	III-IV	11/74 (14.9%)	0/27 (0%)	9.81	1.20–1274	**0.029**	ns		

TLR4-3612TT DNR	III-IV	11/51 (21.6%)	2/56 (3.4%)	6.82	1.88–36.47	**0.002**	12.86	2.47–138.8	**0.001**

IL18-607TT + TG PT	II–IV	39/77 (50.6%)	7/30 (23.3%)	3.08	1.25–8.31	**0.014**	ns		

SSC	III-IV			3.83	1.25–8.51	**0.003**	6.20	1.40–32.31	**0.016**

DNR: donor; PT: patient; SSC: source of stem cells; ns: not significant.
