# Supplementary material for: Impact of Inflammatory Cytokine Gene Polymorphisms on Developing Acute Graft-versus-Host Disease in Children Undergoing Allogeneic Hematopoietic Stem Cell Transplantation
Source: J Immunol Res. 2015 Apr 20;2015:248264. doi: 10.1155/2015/248264 (PMC4417977; doi:10.1155/2015/248264)
Supplement: Supplementary file 1 — SNPs genotyped and minor allele frequencies (MAF) in donors and recipients. [file 248264.f1.docx]

| **Supplemental Table I**. SNPs genotyped and minor allele frequencies (MAF) in donors and recipients.   \| **Gene** \| **alias** \| **dbSNP** \| **Minor/Major Allele** \| **Individual genotyped**  **Recipient / Donor** \| **MAF Recipient** \| **MAF**  **Donor** \| \| --- \| --- \| --- \| --- \| --- \| --- \| --- \| \| CARD15 \| SNP12 \| rs2066845 \| C/G \| 126/128 \| 0.020 \| 0.012 \| \|  \| SNP13 \| rs2066847 \| C/- \| 125/128 \| 0.012 \| 0.021 \| \| ESR1 \| PvuII  XbaI \| rs2234693  rs9340799 \| C/T  G/A \| 121/122  118/114 \| 0.361  0.327 \| 0.415  0.348 \| \| FAS \| -670 \| rs1800682 \| T/C \| 118/114 \| 0.465 \| 0.540 \| \| FCGR2A \| +500A>G \| rs1801274 \| C/T \| 128/128 \| 0.400 \| 0.361 \| \| IL10 \| -1082 \| rs1800896 \| G/A \| 128/128 \| 0.411 \| 0.388 \| \|  \| -819 \| rs1800871 \| T/C \| 118/114 \| 0.250 \| 0.258 \| \|  \| -571 \| rs1800872 \| A/C \| 118/114 \| 0.250 \| 0.263 \| \| IL10RB \| Codon238 \| rs2834167 \| G/A \| 126/128 \| 0.334 \| 0.321 \| \| IL18 \| -137 \| rs187238 \| C/G \| 117/111 \| 0.243 \| 0.270 \| \|  \| -607 \| rs1946518 \| T/G \| 119/114 \| 0.444 \| 0.415 \| \| IL1A \| -889 \| rs1800587 \| T/C \| 117/128 \| 0.288 \| 0.305 \| \| IL1B \| -511 \| rs16944 \| A/G \| 117/128 \| 0.332 \| 0.337 \| \|  \| +3954 \| rs1143634 \| T/C \| 117/128 \| 0.252 \| 0.230 \| \| il1RN \| Intron2 \| rs579543 \| T/C \| 126/128 \| 0.266 \| 0.269 \| \| IL2 \| -330 \| rs2069762 \| G/T \| 119/114 \| 0.380 \| 0.301 \| \| IL6 \| -174 \| rs1800795 \| C/G \| 120/114 \| 0.288 \| 0.325 \| \| MBL2 \| -221 \| rs7096206 \| G/C \| 128/128 \| 0.218 \| 0.179 \| \|  \| -550 \| rs11003125 \| C/G \| 118/114 \| 0.362 \| 0.388 \| \|  \| Codon54 \| rs1800450 \| A/G \| 118/128 \| 0.025 \| 0.132 \| \|  \| Codon57 \| rs1800451 \| A/G \| 117/114 \| 0.164 \| 0.019 \| \| MTHFR \| +677 \| rs1801133 \| T/C \| 123/124 \| 0.425 \| 0.364 \| \| TGFB1 \| Codon25 \| rs1800471 \| C/G \| 128/128 \| 0.063 \| 0.068 \| \| TGFB1R2 \| 1167 \| rs2228048 \| T/C \| 128/128 \| 0.019 \| 0.008 \| \| TLR4 \| -3612 \| rs2770150 \| C/T \| 117/128 \| 0.284 \| 0.293 \| \|  \| -2604 \| rs10759931 \| A/G \| 126/128 \| 0.435 \| 0.353 \| \|  \| -1607 \| rs10759932 \| C/T \| 117/128 \| 0.156 \| 0.158 \| \|  \| +1363 \| rs4986791 \| T/C \| 117/128 \| 0.028 \| 0.043 \| \|  \| +11381 \| rs11536889 \| C/G \| 126/128 \| 0.165 \| 0.127 \| \|  \| +12186 \| rs7873784 \| C/G \| 126/128 \| 0.125 \| 0.178 \| \| TNF \| -1031 \| rs1799964 \| C/T \| 126/128 \| 0.201 \| 0.198 \| \|  \| -863 \| rs1800630 \| A/C \| 126/128 \| 0.157 \| 0.146 \| \|  \| -857 \| rs1799724 \| T/C \| 118/114 \| 0.206 \| 0.169 \| \|  \| -308 \| rs1800629 \| A/G \| 118/114 \| 0.112 \| 0.138 \| \|  \| +488 \| rs1800610 \| T/C \| 128/128 \| 0.198 \| 0.178 \| \| TNFRSF1B \| codon196 \| rs1061622 \| G/T \| 126/128 \| 0.197 \| 0.226 \| \| VDR \| ApaI \| rs7975232 \| C/A \| 118/114 \| 0.422 \| 0.388 \| |  |
| --- | --- | --- | --- | --- | --- | --- | --- | --- | --- | --- | --- | --- | --- | --- | --- | --- | --- | --- | --- | --- | --- | --- | --- | --- | --- | --- | --- | --- | --- | --- | --- | --- | --- | --- | --- | --- | --- | --- | --- | --- | --- | --- | --- | --- | --- | --- | --- | --- | --- | --- | --- | --- | --- | --- | --- | --- | --- | --- | --- | --- | --- | --- | --- | --- | --- | --- | --- | --- | --- | --- | --- | --- | --- | --- | --- | --- | --- | --- | --- | --- | --- | --- | --- | --- | --- | --- | --- | --- | --- | --- | --- | --- | --- | --- | --- | --- | --- | --- | --- | --- | --- | --- | --- | --- | --- | --- | --- | --- | --- | --- | --- | --- | --- | --- | --- | --- | --- | --- | --- | --- | --- | --- | --- | --- | --- | --- | --- | --- | --- | --- | --- | --- | --- | --- | --- | --- | --- | --- | --- | --- | --- | --- | --- | --- | --- | --- | --- | --- | --- | --- | --- | --- | --- | --- | --- | --- | --- | --- | --- | --- | --- | --- | --- | --- | --- | --- | --- | --- | --- | --- | --- | --- | --- | --- | --- | --- | --- | --- | --- | --- | --- | --- | --- | --- | --- | --- | --- | --- | --- | --- | --- | --- | --- | --- | --- | --- | --- | --- | --- | --- | --- | --- | --- | --- | --- | --- | --- | --- | --- | --- | --- | --- | --- | --- | --- | --- | --- | --- | --- | --- | --- | --- | --- | --- | --- | --- | --- | --- | --- | --- | --- | --- | --- | --- | --- | --- | --- | --- | --- | --- | --- | --- | --- | --- | --- | --- | --- | --- | --- | --- | --- | --- | --- | --- | --- | --- | --- | --- | --- | --- | --- | --- | --- | --- | --- | --- | --- |
